# Affect, desire and interpretation

**DOI:** 10.1007/s11098-023-02000-x

**Published:** 2023-07-29

**Authors:** J. R. G. Williams

**Affiliations:** University of Leeds, Leeds, UK

**Keywords:** Desire, Interpretation, Utility, Rationality, Affect

## Abstract

Are interpersonal comparisons of desire possible? Can we give an account of how facts about desires are grounded that underpins such comparisons? This paper supposes the answer to the first question is yes, and provides an account of the nature of desire that explains how this is so. The account is a modification of the interpretationist metaphysics of representation that the author has recently been developing. The modification is to allow phenomenological affective valence into the “base facts” on which correct interpretation is grounded. To use this extra resource within that theory to vindicate interpersonal comparisons, we will need to appeal rational connections between level of valence and level of desire, which this paper sets out and examines.

## Introduction

I prefer running to walking. You prefer walking to running. We plan to meet to talk and exercise together, and our options are a walk or a run. What to choose? One factor that matters: *how much I prefer running to walking* compared to *how much you prefer walking to running*. If you have a very strong preference for the walking option over its alternative, and I have a very weak preference for running over walking, then all else equal, walking is the thing we should do. This reasoning presupposes that the *difference* between the desirability of two options for me can be compared to their difference for you. Such interpersonal comparisons of desire are a familiar part of everyday decision-making involving trade-offs. But an influential group of philosophers and economists insist that such comparisons make no sense at all, and so this ordinary way of thinking about trade-offs rests on a mistake.

This paper is written from the perspective of one who thinks that desires (and desire-differences) are interpersonally comparable, and that our ordinary practice is *not* in error. But I am also a constructor of philosophical theories about what more basic facts *make it the case* that people have desires of this or that strength. A central motivation for the revisionists is perplexity about how such interpersonal comparisons could be grounded in a decent theory of this sort. The task of this paper is to develop a theory that removes this mystery. It will do so by taking my own recent account of how attitudes are grounded (Williams 2020) and improving it by adding an account of the role played by *affective responses* (phenomenological pleasures and pains) in grounding facts about desire.^1^

The stakes are high, because the common-sense way of making trade-offs sketched above is embedded in high theory about how we *should* make trade-offs in societies, and how a “social planner” should make decisions for society as a whole. In general, aggregating individual preferences among a group can matter in many contexts, and how we can think of this is shaped by what kind of interpersonal comparisons make sense.^2^ For example, suppose the government faces a choice whether to raise taxes to invest in early-years education provision, or not. Setting aside technical implementability, political feasibility, deontological constraints and the like, can we articulate some standard which determines whether the move would be in the collective interest (understood as an aggregate of preference-satisfaction) or not?^3^ A utilitarian characterization of the standard is the following: every citizen either prefers education investment over status quo taxation, or the reverse, or is indifferent. In each case, there is a fact of the matter *how much more they want* the first option over the second (positive for the first group, negative for the second group, zero for the third). The overall utilitarian recommendation, then, is determined by adding up all these preference-differences, and seeing whether the output is positive (favouring education investment) or negative (favouring status quo taxes). For this utilitarian account to make sense at all, desires (specifically, desire-differences) must be interpersonally comparable. This is not an isolated problem for the utilitarian account of how to aggregate interests. For example, Rawlsians tell us that a social planner should take action that prioritizes the worse off—but facts about who is worse off, preference-satisfaction-wise, depend on facts about relative levels of desire. More broadly, this entire area of theory is shaped by the Arrow impossibility theorem, and this is, in effect, an argument that any recipe for determining the collective interest on the basis of individual desires is either structurally unreasonable, or depends on individuals’ degrees of desire being comparable.^4^ If the argument of this paper is correct, then we escape these impossibility results because desires are indeed interpersonally comparable.

The plan is as follows. The first half of this paper concerns the problems that interpersonal comparisons pose. In Sect. 2, an influential but toy theory of how degree of desire gets determined is described, and it is shown how that leads to the incomparability. Section 3 presents some instances of desire-comparability facts that we would need to get right. In Sect. 4, I set out my recent interpretationist metaphysics of mental content, and Sect. 5 develops a prima facie case that, while it would ground desire comparisons, it would ground the wrong ones. The second half of the paper is devoted to solving these problems. Section 6 lays out an account of phenomenological affective states, and Sects. 7 and 8 examine two accounts of their normative links to propositional attitudes. Section 9 describes how this material, added to the interpretationist theory, grounds the correct desire comparisons.

## Part I: the problem

### Warm-up: the problem as it arises in a simplified setting

This section describes how a particular theory of *what desire is* will entail interpersonal incomparability. This theory at issue in this section is a toy model that helps illustrate the underlying issue in a clean way, and sets us up for seeing how fully-fledged theories defended in the recent literature are afflicted by related problems.

On to the toy theory. We consider a space of possible interpretations of the agent. Each interpretation is an assignment of degrees of belief (probabilities) in propositions and strengths of desires (utilities) across propositions. We say that if the choice disposition is to choose O out of O_1_…O_n_, then an interpretation fits with (structurally *rationalizes*) this choice-disposition iff it assigns an *expected utility* to O that is at least as high as any of the O_i_. The next step is to say which of the interpretations are correct, and we do this by imposing one or more filters on the candidate interpretations. The interpretations which survive the filtering are the correct ones. Here is one candidate to be THE filter: knock out any candidate interpretation which doesn’t fit with the choice dispositions.

Here’s the central observation: if we take an interpretation (probability-utility pair) that fits with a specific choice disposition, and transform it by boosting all the utilities by some constant, it still fits with that choice-disposition. The expected utility of each option in the relevant set goes up by that constant, but a uniform boost (or subtraction) doesn’t change their ordering, and the only question that the filter cares about is whether the chosen option sits on top of that ordering. If you multiply all the utilities by a positive factor, essentially the same thing happens—gaps could widen or shrink, but the ordering of the options is preserved. Whether an agent is represented as choosing the best out of a good set of options, or the least-bad from a bad set, doesn’t matter at all. In sum: if any one interpretation from our space of interpretations passes this filter with respect to a given set of choice-dispositions, so do all the others that differ from it in the ways just mentioned.^5^

Unless some further filters are added, therefore, this story about what fixes how strongly a person desires a thing can never, in principle, narrow the facts down to a single interpretation. It always selects a whole *class* of interpretations, where within that class we have arbitrarily boosted and expanded assignments of degrees of desire. In this situation, we say that this metaphysics of preference makes it *indeterminate* what any given person’s level of preference is, and *indeterminate* what the size of the gaps in preference are, with the filtered desire-assignments being “precisifications” of the indeterminacy.

Now, what if we’re interpreting multiple individuals at once, Ann and Bob say? Well, our interpretation of Ann better rationalize her choice-dispositions, and our interpretation of Bob better rationalize his. But once we’ve rationalized each individual’s choice-dispositions, there’s nothing in the story that demands we “coordinate” the interpretations. So if you pick any one interpretation from the set of “correct” interpretations of Ann, and any interpretation from the set of “correct” interpretations of Bob, then you get a sequence of interpretations of Ann and Bob that pass the only tests we have for being a correct pair of interpretations for Ann and Bob together. This destroys any chance of interpersonal comparability of desire. If there were to be fact of the matter that Ann desires walking more than Bob does, for example, there would minimally have to be one correct interpretation of Ann and one of Bob on which Ann’s utility for walking, *a*, is greater than Bob’s, *b*. Now choose some constant *k* > *a*—*b*. By the previous intrapersonal indeterminacy result, we can boost all Bob’s utilities by *k*, and the result is another interpretation that passes all the filters for being a correct interpretation of Bob. But now putting together the original interpretation of Ann, and the boosted interpretation of Bob, and we have a correct sequence of interpretations on which Ann’s utility for walking is *a*, which is less than Bob’s utility for the same thing, *b* + *k*. That is: among the correct sequences of interpretations is at least one sequence on which Bob desires walking more than Ann does. Reductio! So on this toy theory of what fixes facts about strengths of desire, there is no fact of the matter about interpersonal comparison of degrees of desire (similar arguments will show the same for interpersonal comparison of utility differences, and so forth).

## Instances of desire-comparability

Revisionist theorists see the indeterminacy results above as discoveries, rather than problems for the metaphysics of desire that generates them. I take the opposite view, and so view such results as helpful tests for seeing when a metaphysics of desire needs supplementation. The task of this section is to construct some focal cases which illustrate the kind of comparisons that those of my persuasion will think a metaphysics of desire *should* vindicate. This is necessary because the test for success will not just be to vindicate *any* desire comparisons; it is to vindicate *the right* desire comparisons. I’ll soon be arguing for changes to my own theory on the grounds that it fails this second test.

To build the case, start with a very normal scenario. Let Ann, on Monday, be someone who has preferences and a life history much like you—but with the following (possible) change. She absolutely hates walking, and hates running only a little less. You can, I think, simulate her psychology with just a slight modification to your motivational set.

Now let’s imagine Ann’s almost-doppelganger, Ann*. She, on Monday, also has preferences and a life history much like you—but with the following change. She absolutely *loves* walking, and *loves* running even more. You can, I think, simulate this alternative modification to your motivational set equally well.

On Monday, I say, Ann’s degree of desire to go running is less than Ann*’s degree of desire to go running. If there are facts about interpersonal comparisons of desire, this is one of them.

Let’s go through the week. Ann's mood gets darker over time. She finds less joy in companionship, in work, in everyday amusements. Everything is looking worse to her—except for running and walking, which were of course, already very bad by her lights. Eventually, she comes to hate almost everything approximately at the same level as she always hated walking and running. We stipulate: she continues to choose rationally—choosing to do what maximizes utility for her, which now means she consistently opts for the least-hated option. Sometimes, even running now seems the best of her available options.

Over the week, Ann*'s mood gets brighter and brighter. She finds more joy in companionship, work seems inspiring, and everyday amusements increasingly delightful. She continues to choose rationally—only now, sometimes the walking or running she still loves is outcompeted by other options, and only sometimes does she find herself indulging in her old passion.

By the end of the week, the *pattern* of Ann and Ann*'s desires for outcomes completely match. On Friday their utilities across outcomes are transformations of each other’s—differing only because Ann*’s are systematically “boosted” compared to Ann’s.

Again, if there are facts about interpersonal comparisons of desire, this comparison between Friday-Ann* and Friday-Ann is one of them.^6^

Because of their similarly-patterned desires for outcomes, combined with their similar histories leading to similar beliefs, Friday-Ann and Friday-Ann* will be disposed to make the same choice in many decision situations. If faced with running vs. TV, they both, say, opt for running. For Ann, this is a matter of choosing the least-bad option, for Ann*, choosing among delightful options; but the result is the same. However, this is not universal: Ann and Ann* have different histories, and unless they suffer complete amnesia, that will show up in differences in their beliefs, and in any behaviour that depends on this. For example, they would choose to utter different words when asked to report about whether they like TV better or worse than they have done in the past; Ann will say ‘worse’ and Ann* ‘better’. So the residue of past differences means they don’t have entirely similar choice-dispositions.

The final instance of comparability that I want to introduce cleans up even this source of difference. I submit that if the two patterns of desires-for-outcomes that Friday-Ann and Friday-Ann* instantiate is possible, it’s also possible that another agent, Bob, starts out on Monday with a set of desires that matches Friday-Ann’s, and possible that another, Bob*, starts out on Monday with a set of desires that matches Friday-Ann*’s. We may stipulate that Bob and Bob* have life histories that are as similar as possible, consistent with this.

If there are facts about interpersonal comparisons, then it will be a fact that Bob has generally lower degrees of desire in running (and most other things) than Bob*. But now it will be extremely difficult to locate this difference in any difference in choice-dispositions, because not only do they have the same pattern of degrees of desire over outcomes (just differing by a boost) the *beliefs* that mediate these desires to determine their decisions are relevantly the same. They aren’t disposed to report a slide or raise in degree of desire over time, for example, unlike Ann and Ann*. I think it’s plausible that Bob and Bob* can have the same choice-dispositions.^7^ This is the case I’ll be mainly focusing on going forward, and the test for success of a theory will be whether it can predict the right thing in the Bob/Bob* case.

## From the toy theory to radical interpretation

If interpersonal comparisons of desire are real, then something has gone wrong with the toy theory sketched in Sect. 2, because it fails to vindicate the comparability facts illustrated in Sect. 3. Some filters—either at the level of individual interpretations, or of the interpretation of sequences of individuals—must have been left out the story. This section considers a recent theory of content—in inspiration an elaboration of the toy model—and in later sections I will argue it suffers related difficulties, and use this to propose amendments to it.

The focus will be a version of Radical Interpretation—the version that I’ve set out and defended in recent work (Williams 2020). This is a story about how desire (and belief) are fixed. It is of the same general *style* as the choice-based filtering story of Sect. 1, but with more bells and whistles. We again start with a space of interpretations, though this time we model these as interpretations that map particular inner states of a person to attitude-strength-proposition triples. The account endorses the following pair:

- A person *x* has a desire of strength *d* with content *p* iff the correct interpretation of *x* maps some inner state *s* of *x* to the triple < desire,*d*,*p* > .
- I is the correct interpretation of a person *x* iff I is the interpretation that best rationalizes the way *x* is disposed to *act* given *x*’s *evidence*.

The second line is supposed to give a “real definition” of correct interpretation, so that in the first line the direction of explanation can be taken to flow from right to left across the biconditional.

We may divide the active elements of the crucial second line into two. There are some facts about the target *x* that are taken for granted—the *basis* on which interpretations are selected as correct. These include facts about how the agent is disposed to act, and what their evidence is. Those are intentional descriptions of aspects of the agent, so this story is not an account of how the representational is grounding in the non-representational world. It is a story about how to transform a certain kind of source intentionality (perception, intention) into others (belief, desire).

Alongside the basis of interpretation, there’s the selection-story—the way that interpretations are selected as correct, given the basis. According to the above line, this is a matter of maximizing the overall rationality of the target. So those contrastive intentions had better make sense in light of the beliefs and desires attributed, and the beliefs, in particular, had better update in the right way given the perceptual evidence. A major choice-point here is the notion of rationality we appeal to. My proposal is to deploy a very thick notion of rationality. On this understanding, interpretation-selection is reason-maximization—we seek an interpretation that maximizes the normative reasons possessed by the agent for their beliefs (which points to justification-maximization), and the normative reasons possessed by the agent for their acts/intentions (which points, inter alia, to morality-maximization). We can assume that part of maximizing reasons in this sense is ensuring that the agent is sufficiently structurally rational (e.g. has logically coherent beliefs, and satisfies means-end constraints between desires and intentions). Maybe that comes in because you need a certain level of structural rationality in order to count as basing your beliefs or intentions on reasons in the first place; or maybe it’s because we have reasons to be structurally rational.^8^

The radical interpretation model diverges from the toy theory of Sect. 2 in a number of respects. In the base we now have evidence, as well as choice-dispositions. In the selection story, we are now talking about maximizing rationality, rather than fitting all the choice-dispositions. And while “fitting choice dispositions” in the toy theory of Sect. 1 was a matter of means-end structural rationality (i.e. having the chosen option be the one which maximizes expected utility) radical interpretation opens the floodgates to all sorts of appeals to normative requirements. The question for us is: are these changes enough to avoid the threat of interpersonal incomparability of desire? That threat was derived from an underlying *intrapersonal* indeterminacy in levels and scales—the lack of any way to select between interpretations that differ from one another solely by a boost or expansion of utilities/degrees of desire. Does radical interpretation, as formulated here, make any advance?

## Radical interpretation may ground the wrong desire-comparisons

This section presents a prima facie case that the radical interpretation theory of Sect. 4 will not predict the right interpersonal comparisons. A loophole will be left open, however, which can be exploited by the modified account, to be developed in the second half of this paper.

Several of the ways that this version of radical interpretation differs from the toy theory don’t help at all with our problem. The adoption of “maximizing rationality” as the mode of interpretation-selection doesn’t do anything to resolve the indeterminacy, which after all manifested even in hypothetical, perfectly structurally rational agents. The diachronic aspect of radical interpretation (interpreting all time-slices of an agent, rather than choice-dispositions at a snapshot in time) does nothing to filter out uniform utility boosts in the Bob/Bob* case, where are problem is to rule out a systematic boost across all time-slices of an agent. The appeal to *inner states* rather than stages of the person as the locus of interpretation doesn’t help resolve our problem at all.

I submit that the *only* thing here with the potential to resolve the difficulty is the thickening of the conception of rationality. If my 2020 account, as it stands, entails interpersonal comparability of desire, it’ll be because it asks that the beliefs attributed be *justified* by the interpretee’s evidence, and that their choices be *based on good reasons* rather than simply hang together coherently.

The most direct way of leveraging this would assess directly how (substantively) rational the degrees of desire an interpretation attributes to the agent are.^9^ This is based on the anti-Humean thought is that there are *reasonable* and *unreasonable* strengths and patterns of desires to have in particular propositions. Having a final desire for a saucer of mud is unreasonable, says the Anscombian anti-Humean, since there is no real value to saucers of mud. Likewise, the Nozickian utility monster is an agent whose desire-gaps between having one level of resources and the next are ever-increasing; and the anti-Humean says: these are unreasonable desire-gaps to have. Once we allow anti-Humean resources in, they can do a lot of work, since even desires for things of value (e.g. the pleasant companionship of friends) should, it seems, be kept in proportion by the substantively rational agent. I slightly prefer granary bread over white bread for tea. I would be substantively irrational, however, if the gap between my levels of desire for these things were as wide as the difference between my present levels of desire to be sitting in a warm front room, vs. being tortured to death. Thus, boosts and expansions of desires can easily transform a reasonable, well-proportioned psychology into an unreasonable one. Reason-maximizing radical interpretation will favour the former over the latter, breaking the symmetry even if both *structurally* fit with all choice-dispositions, evidence, and so forth, and even if the agent is *epistemically* perfectly reasonable.

Here’s a simple model of how to think of the appeal to substantive rationality in interpretation. Just as there are strengths of beliefs you should ideally have in your situation (maybe they should be aligned to the truth values, or aligned to the evidential probabilities), there are strengths of desires you should ideally have in outcomes (maybe they are aligned to the objective value of the outcome). Various filters (e.g. means-end rationality, coherence) can winnow down the space of interpretations of our target, but after all else has been factored in, if multiple options remain, we should favour those that are “most similar” to the rational ideal.^10^

Anti-Humean constraints on desires (in the context of reason-maximizing radical interpretation) can rebut the case for incomparability from Sect. 2. Whatever structure we pack into the gradation of values, we can argue for in desires. For example, if there are objectively neutral states, objectively positive ones, and objectively negative ones, then we can say that Ann is getting things *right* when she finds the objectively negative-for-her prospect of walking-and-talking undesirable (a strength of desire < 0). And an interpretation that assigns this to her will be favoured (all else equal) over one which boosts all her assigned utilities sufficiently to give her a utility of + 1 in walking-and-talking, even if the latter can structurally rationalize all her choices, given her evidence.

This looks initially like progress: at least for anti-Humeans, there’s no cogent argument left for interpersonal desire incomparability.^11^ However, the question is whether this combination can ground the correct comparability facts, with the Bob/Bob* case from Sect. 3 an instance. And there’s a prima facie case that it will not. Bob and Bob*’s respective life-histories are by construction very similar. In particular, the courses of their sensory experiences can be identical; the worldly results of the choices they make will be the same. The salient threat, then, is that normative theory predicts that they have the *same* reasons for belief, and the *same* reasons for desire. If those are the normative facts, then my 2020 reason-maximizing version of radical interpretation will indeed generate desire-comparisons between Bob and Bob*, but it will say, falsely, that they have *identical* levels of desire, because the most *reason-maximizing* interpretation that rationalizes their choice-dispositions is the same for them both. If this is where we leave things, then we have moved from frying pan to fire, and desire-comparisons serve just as much as an objection to radical interpretation as to the original toy theory.

I think the move from the toy theory to interpretationism is progress—it is just that it we need to put in more work before the job is done. I take up this task in Part II.

## Part II: the solution

Let us take stock: the position of this paper is that there are facts about desire-comparisons, and a metaphysics of desire is only adequate if it can ground facts of this kind. It must also, of course, ground such facts *correctly*. The toy theory of Sect. 2 leaves interpersonal comparisons completely indeterminate, and that was no good. Radical Interpretation, as spelled out in Sect. 4, has extra resources and may well predict that degrees of desire are comparable, but we’ve seen a prima facie case that it predicts the wrong comparisons, and that’s no good either. This second part of the paper moves into a constructive mode, and shows how by adding certain phenomenological facts about *affect* to the interpretationist’s base, we can resolve our puzzle.

## Characterizing affective valence

In his forthcoming review of *The Metaphysics of Representation*, Adam Pautz notes that I say little-to-nothing about the rational significance of the *pleasure taken* in a job well done, or the *unpleasantness* of a cut finger. He suggests that appeals to these affective experiences are a resource that I could use to constraint the selection of the correct (most rational) desires:

“Sally has a range of experiences with “valence”: pleasures, pains, gustatory experiences, feelings, and emotions. … Just as it may be a basic fact that visual experiences give Sally reasons to believe various things, maybe it is just a basic fact that certain affective experiences …. give her a reason to desire certain things to varying degrees. If so, then the “best interpretation” will be one that tends to assign her desires that are “reasonable” given her affective experiences.” (Pautz 2023, p 317)

Developing this basic idea and connecting it to interpersonal comparability will take the rest of the paper. This section sets out the functional-metaphysical picture of affect I will work with. The next two sections articulates normative principles relating affect and desire. By using these, facts about phenomenological affect will, in the context of Radical Interpretation, ground desire comparisons.

Here’s an illustration of the basic idea. I love walking-and-talking. You hate it. If interpersonal comparisons of levels of desire make sense at all, then the lover must surely desire walking-and-talking more than the hater does. But (propositional) loving and hating are not just different states of desire—they also differ phenomenologically. Certain “affective” feelings that are part of each state. I take *pleasure* in walking-and-talking, whereas you find it *unpleasant.* This contrast carries over to the Bob and Bob* case I constructed in Sect. 3. The intuitive case was this: while Bob *hates* running (and hates most other options, to a greater and lesser degree) Bob* *loves* running (and loves most other options, to a greater and lesser degree). Bob and Bob* were stipulated to be as similar as possible in total life histories, consistent with these stipulations. Now, I submit that this will mean that will have identical courses of *sense-experience*, but there will be an aspect of their life-histories in which they differ: they have different courses of *affective experience*. Bob’s is marked by displeasure, and Bob*’s by pleasure.

In order to spell out the idea in detail, we need a working model of what the relevant contrast in positive and negative affective experience (“valence”) is and how it works.

The model of valence I will use has the following two key characteristics:

1. Phenomenological: different strengths of valence are different phenomenological states.
2. Responsiveness: valenced phenomenology is always produced in response to mental states of some independently characterizable kind (a judgement, a sensation, etc.).

Eden Lin’s (2020) theory of pleasure incorporates (1) and (2). Pleasure, for Lin, is a certain type of phenomenal experience which can be part of (or an aspect of) a broader mental state. It can be an aspect of a mental state which contains as a proper part a sensory state, and it can be part of the mental state we are in which contains as a proper part a representational state: judgements, perceivings, etc. Terminology: when a mental state M and a valence state V are proper parts of a third mental state, I will say that V is attached to M.^12^

Now, if valenced phenomenology is to be *occurrent*, and produced in response to a mental state, you might assume that the mental state that produces it would need to be occurrent. But that doesn’t follow. The standing state of a room being filled with gas, in the presence of a spark, produces an explosion. Just so, a standing belief that one is a failure, when attended to, can produce negatively valenced phenomenology. When one is disposed to feel valence V upon attending to standing belief B, I’ll assume that B and V will count as parts of a standing mental state B + V—in the terminology above, valences can be attached to standing states of belief, just as much as to sensations, perceptions or judgements.

As Lin says, this does not imply that affect is strictly part of the sensory or representational state—it is mental paint that is added over and above whatever prompts it. This picture of superadded pleasure (which I am identifying with positive valence) allows us to say that two people could have duplicate sensory states (say, of eating hot chilli) and one find it pleasant, the other unpleasant. It also makes clear that the affective phenomenology need not be located *within* sensory phenomenology—taking a hot bath has a distinctive haptic sensory feel, and eating hot chilli has a completely different sensory feel, with no sensory similarity at all. On the Lin-style account, we should not worry about this: pleasure is phenomenological, but it is no part of sensory phenomenology. After all, the phenomenology of positive affect can attach in the same way to states that have no sensory phenomenology at all, such as a belief or judgement.

Lin’s picture dovetails with the theory of affect advocated by Lisa Feldman-Barrett, on the basis of neuroscientific research (; b). Feldman-Barrett, like Lin, thinks of affective feelings as a fundamental kind of phenomenology, and defends a two-dimensional “circumplex” model of affect, on which valence (positive or negative) is one dimension, and activation/arousal the other. Each basic affective feeling corresponds to some point in this grid—the feeling of excited anticipation being high arousal/positive valence; a feeling of relaxed happiness being low arousal/positive valence; a feeling of anxiety high arousal/negative valence; and a feeling of depression or despair low arousal/negative valence. Her hypothesis is that affective feelings are in fact interoceptive sensations—that is, low-resolution sensations of our inner body state (hormone levels, heart rate, gut activity, etc.). As occurrent thoughts and inner/outer sensations impinge on us, our nervous system will change the body state in preparation for imminent or less imminent action, and this shows up in consciousness as an affective response to the mental episode in question. Lin’s complex mental state of taking pleasure in hot chilli would be the complex state of eating hot chilli plus feeling pleasure (i.e. sensing a body state that shows up as pleasure) in response.

Importantly, the two-dimensional *space* of possible affective feelings is common to all of us, I will assume, whatever our individual differences in the specific affect we attach to specific episodes. There is always a fact of the matter about whether how similar or different (how close within the affective circumplex) the affect I attach to a particular hot-chilli sensation is to the affect you attach to it. This assumption is analogous to assuming that the colour solid in which our respective colour experiences are located is the same. It will be this interpersonal comparability of phenomenology that grounds the interpersonal comparability of desire, on my telling.

That affect is a big part of our lives is obvious. But what exactly is the role that it plays? My understanding is that, prior and independent of the connection to sophisticated mental states of belief and desire, it shapes our behaviour. Suppose you’ve eaten a first piece of a chocolate bar, taken pleasure in it, and have decided to save the rest for later. As you continue to work on your essay, you turn back to the chocolate wrapper to find a piece missing. You picked up and ate the next piece of chocolate (against your consciously-chosen plans) ‘absent-mindedly’. This is not rational/irrational *action*, but arational *activity.* Much of our agency consists in moving about and interacting with the world arationally—avoiding obstacles, scratching itches, consuming chocolate—in a mode that’s under the radar of our beliefs and desires. Valence directs this under-the-radar activity, with activities that promote states of positive valence promoted, and activities that promote states of negative valence suppressed. That gives us a functional fix on the difference between the different signs of valence, and also gives a functional fix on the relative strengths of valence. So this is a third assumption, alongside the (interpersonally comparable) phenomenological nature of affective feelings and their responsiveness:

1. Activity-directing: the functional role of valence includes directing activity

(2) and (3) together give a high-level picture of the functional role (1) of valence within organisms—its causes and consequences. This is quite a committal picture, though a prima facie plausible one in line with best recent research in the area. The reader might wonder about further questions: what *is* affect, fundamentally? A primitive dualistic quale? The instantiation of the mentioned functional role? An imperative to seek out or avoid the attached mental state?^13^ Something else entirely? But these are not issues I will take a stand.

## (Putative) narrow-scope normative links between affect and desire

Affect is something that differs among the individuals in the examples of interpersonal desire comparisons from Sect. 3. In this section, I examine one way of leveraging these differences within radical interpretation to get it to predict the right results (this would constitute a rebuttal of the prima facie objection to interpretationism from Sect. 5). I will not be recommending this route, since I don’t accept the particular auxiliary normative hypotheses involved. The next section will develop a different route involving a different auxiliary hypothesis that I do endorse.

It doesn’t seem to be a coincidence that, normally, people want the things associated with positive affect and don’t want things associated with negative affect. Pautz sketches one way of thinking about this. He says “affective experiences …. give [a person] reason to desire certain things to varying degrees”. I think he has the following picture: one undergoes a certain experience (say, of eating hot chilli). This experience is accompanied by positive affect (pleasure). The fact that one has experienced the hot chilli eating *with pleasure* gives one reason to (intrinsically) desire hot chilli. This suggests a general picture whereby the fact we have affective experiences with a certain content gives us (narrow scope) reason to (intrinsically) desire that content obtain, much as a perceptual experience with a certain content gives us reason to believe that the content obtains.^14^

Narrow-scope principles of the sort Pautz posits would very much help my interpretationist tackle the focal case from the first part of this paper. Bob and Bob* had different courses of affective experience, remember—Bob’s life history is marked with very negative affective valence attached to his experiences, while Bob* attaches highly positive affective valence to the analogues. On the Pautz-like account, the idea would then be that while his affective experiences give Bob reason to have negative degrees of desire in each possible outcome, Bob*’s affectively boosted experiences give Bob* reason to have positive degrees of desire in those outcomes. The identical choices that Bob and Bob* are disposed to make leaves them with a whole range of possible desire-assignments that would equally well (structurally) rationalize those choices. But there is now a tie-breaker: you maximize substantive rationality by picking the assignment for Bob (negative desires) which his course of affective experience gives him reason to have; and you do the same for Bob* by picking the assignment (positive desires) which his *distinct and boosted* course of affective experience gives him reason to have.

Job done? No. I submit that affective experiences do not give us reason to desire things to a degree that matches their valence. It seems to me that this would be analogous to saying that our *felt confidence* in a proposition gives us reason to believe it, to a matching degree. It seems to me a very common experience that we realize that we are disposed to attach negative valence to something (e.g. someone giving us honest feedback on our work) where that thing is what we *want.* The appropriate response in this case is not to adjust desires downward; it is to relax and let the frustration go, to try to achieve a state where the affective valence matches the degree to which the state in question is desired. That’s similar to the situation with felt confidence: sometimes perhaps felt confidence is more in line with what one should believe than one’s actually functionally-identified degree of belief, but often the reverse is true. If there were Pautz-style narrow-scope principles around here, affective over- and under-reactions, whether positive and negative, would be something like the desire-analogue to perceptual illusions. This rings false to me.

Furthermore, even if Pautz’s principles were true, it’s not obvious that *all things considered* the reason-maximizing interpretation of Bob gives him very negative desires and the most reason maximizing interpretation of Bob* gives him very positive ones. For even if both have valence-given *pro tanto* reasons to have desires of those respective intensities, this might be outweighed by other *pro tanto* reasons, based on the intrinsic qualities of the things desired, which are the same for both Bob and Bob*. So the puzzle isn’t conclusively solved.

I conclude that those who agree with Pautz and disagree with me about the narrow-scope normative significance of valence have a possible strategy for patching interpretationism. But there is a better way.

## Wide-scope normative links between affect and desire

The position of this paper is that differences in an agent’s affective profile explain the interpersonal desire comparisons, via normative connections between affect and desire that take *wide scope.* This section sets out and explores the normative links in question; the next will connect this to interpretationism.

I posit a rational tie between affectively laden *beliefs* and *intrinsic* desires.^15^ Unlike Pautz, the normative link I’ll explore here will be a wide-scope and structural, rather than narrow-scope and substantive. Here it is:

One ought to be such that:

One has a valence of a certain sign and strength *k* attached to the belief that *p*, only if one has an intrinsic desire of the same sign and strength *k* in *p*.

This principle is supported by, though does not entail, the following two-part normative picture. First, Kagan’s (2009) thesis: a person’s well-being consists in her enjoying the good (or good-for-that-person), where “enjoying *q*” can be understood in the present terms as attaching positive valence to the recognition that *q* obtains. So one *should* find pleasant the things that are good (for you): on Kagan’s analysis, doing so maximizes your well-being, holding fixed the set of good and bad things you possess. Second, an anti-Humean principle about desire: you should intrinsically desire states of affairs that are good (for you). Put these two thoughts together: if you’re feeling the right feels, and desiring the right things, then you’ll be finding things pleasant iff they’re good (for you) iff you intrinsically desire them. So if the two “internal” states here—the finding-pleasant, and the desire-state—are not aligned, then that is a *guarantee* that something is not as it ought to be. The wide-scope normative thesis above is one candidate articulation of a resulting connection between internal states—a requirement whose violation guarantees error.^16^

Let me now turn to some of the features of the wide-scope principle I’ve proposed. The first crucial thing about it is that it is wide scope. Consider instances where one takes pleasure in the wrong things: the suffering of others, perhaps. I don’t think one should desire that others suffer, and I don’t think that the fact that you enjoy it gives you a reason to desire that state of affairs, and certainly not a reason to intrinsically desire it! And the wide-scope principle by design avoids such predictions. There is indeed a wide-scope demand is to match affect to intrinsic desire. But the right way to meet this is to come to have a different affective response to the suffering of others.^17^

Note also that this wide-scope principle ties affect to level of *intrinsic* desire, rather than level of *instrumental* desire. One and the same state of affairs (or proposition describing that state of affairs) can be both intrinsically desirable, a good-making feature of the world, and have low utility. By the same token, a state of affairs can be intrinsically undesirable or neutral, and have high utility. In my understanding, intrinsic desirability is a matter of how the utility of *worlds* is determined, as a function of what goes on within them. Utility of worlds plus belief rationally fixes the (expected) utility of propositions in general. Instrumental desire is a matter of *how good the news is that a proposition is true*, and intrinsically good-making features of a world can be sign of bad things, and vice versa.

Let’s test this out by seeing how the wide-scope account handles cases where certain facts are evidence for further facts:

- I see the red flag flying over the castle, and infer that our troops have taken it, and rejoice. The account above would have a problem if this case was one where I rationally take pleasure in the red flag flying, since what colour flags fly where is something I’m intrinsically indifferent to. But I infer from the red flag flying to something else: I come to believe that I have achieved victory and glory (it’s a sign my troops have captured the castle). And I intrinsically care about victory and glory, and so take pleasure in the belief I’ve achieved this. The account does not predict that I take pleasure in the belief that a red flag is flying, but it does predict that this belief is only one step removed from a state in which I will take pleasure.
- A monarch loves their general, and intrinsically desires the general live a long and happy life. They love their kingdom more, and they really want to see it survive. Suppose the battle has progressed so that (the monarch knows) the kingdom will be saved iff the general sacrifices themselves. The monarch receives the news that the general has been killed. This is a state of affairs that intrinsically the monarch does not want to obtain, but it is also something that indicates the obtaining of a different state of affairs that the monarch very much wants to obtain. The valence-desire connection predicts the following. If the monarch’s psychology is as it ought to be, then, first, the monarch forms the belief that the general has been killed, and this has negative affect—it saddens them; second, the monarch infers from this that the kingdom has been saved, and this has positive affect—it gladdens them. They take pleasure in the saving of the kingdom, and displeasure in the death of the general. Very plausible!^18^

The wide-scope principle is restricted to states of affairs that the agent believes obtains. This has a couple of striking consequences for aversive mental states:

- Suppose that an agent has an intrinsic desire to know whether *p*. The wide-scope principle will be violated unless they have negative valence associated with the recognition that they are uncertain whether *p*. The wide-scope principle does not require, on pain of irrationality, that negative valence is attached to uncertainty itself, but only that this must attach to any higher-order belief that one is uncertain.
- The same goes for a paradigm of strongly aversive mental state: pain. If one has an intrinsic desire to not be in pain, then the wide-scope principle requires, on pain of irrationality, that the higher-order belief that one is in pain is negatively valenced. It is silent on whether the painful sensation itself is negatively valenced.

These are features and not bugs. In neither case do I rule out the uncertainty or the pain itself being negatively valenced—indeed, presumably they will be, and the functional role articulated in the last section says that such valences will arationally direct activity towards avoidance of such states. But the wide-scope principle simply doesn’t have such cases within its scope.

It might seem that the negative affect attached to pain should—for a rational creature—rationally influence its deliberation, and not just arational activity. Nothing I’ve said entails otherwise, since I don’t say the principle above is the *only* rational connection between valence and desire. For example, I think the following cousin of the earlier principle is also plausible:

One ought to be such that:

One has a valence of a certain sign and strength *k* attached to the awareness that *p*, only if one has has an intrinsic desire of the same sign and strength *k* in *p*.

Physical pain might be identified with *awareness* of certain kinds of bodily disruption. Uncertainty might be identified with *awareness* of lack of polar belief. Conditional on those identifications, the awareness-form of the principle would tie the negative valence attached to pain and uncertainty themselves to intrinsic desires against the bodily disruption or the lack of polar belief.

Let me finish this exploration of the wide-scope normative principles with two puzzle cases^19^:

- Suppose Sally strongly intrinsically desires to ameliorate intense suffering (she adopts a career that is centred around this). But her belief she has achieved her desire, in any given instance, is not attached to very positive valence—indeed, it would seem inappropriate given that the person in question is still suffering, albeit at a lower level.
- Sally finds climate change and the expected devastating human cost *strongly* undesirable. Let’s suppose she dedicates much of her life and resources to campaigning against it. Someone cutting in front of her in the line, on the other hand, is something she desires not to occur, but the strength of this desire is vanishingly small in comparison—she doesn’t invest resources in a campaign against queue-cutting. Yet normal human psychology is to have a very intense negative-valence affect to cutting in line (it makes one angry) while climate change wouldn’t produce anything like the same intensity of affect.

To the first of these puzzles I respond: the person does not *positively* desire that the world contain the ameliorated suffering, any more than they *positively* desire that the world contain the intense suffering. They appropriately attach negative affect, therefore, to the belief that the person has the medium level of suffering, but are also strongly motivated to take actions that avoid the even less desirable world of high levels of suffering. They can appropriately attach positive affect to personally having made a positive difference in the world—making it less undesirable. But that positive affect is apt pleasure in right action, not inapt pleasure directed at the state produced.

To the second of these puzzles I respond: we can explain some of the “mutedness” of affect associated with climate change by appeal to the second dimension of what Feldman-Barrett calls the affective circumplex: the immediate, actionable observation of someone cutting into the queue produces a high activation negative valenced state, whereas an abstract, future, bad state of affairs is unlikely to be highly activating. A worry remains: the affective *valence* attached by Sally to the realization that climate change will happen (let’s stipulate) is not many multiples worse than than the queue cutting. To this residual worry I say: it is an advantage of the wide-scope formulation of my constraints that they allow us to diagnose irrationalities that spring from our affective states being misaligned with our strengths of desire. We have strong negatively valenced responses to things that don’t, on reflection, matter that much. Rationally, we shouldn’t react so strongly. It’s also a familiar fact that we often lack the emotional range to react as strongly as we might to abstract, depersonalized tragedy. For many of us, that’s also correlated also with a low functionally-revealed-motivation to take steps to mitigate abstract, depersonalized tragedy. These are not the most admirable human traits, but they are familiar ones. If we manage to correct one but not the other error (as stipulated in the case above), then the predictable result is a kind of structural irrationality reflecting the partial nature of the success.

## Grounding interpersonal comparability in affect and normative links

Our puzzle from the first part of this paper was that theories of what grounds desire didn’t have a good way to ground interpersonal desire comparisons. In particular, my own recent theory of radical interpretation, while it made progress in allowing desire-comparisons in principle, predicted the wrong desire-comparisons. So far in this second part of the paper, we’ve set out an account of affect, and a wide-scope normative principle that links it to desire. We now close the loop and show how to modify radical interpretation to reflect these facts. In doing so, we can generate the right desire-comparability predictions.

The defining theses of radical interpretation given earlier were the following:

- A person *x* has a desire of strength *d* with content *p* iff the correct interpretation of *x* maps some inner state *s* of *x* to the triple <desire,*d*,*p*>.
- I is the correct interpretation of a person *x* iff I is the interpretation that best rationalizes the way *x* is disposed to *act* given *x*’s *evidence*.

I want to refine this by extending the basis on which the interpretation is selected. In my 2020 account, in advance of interpretation, we help ourselves to quite a rich base level of facts, which are appealed to in characterizing correct belief-desire interpretation. To this basis, I now add an extra layer of assumptions: facts about what valence the subject attaches to the interpretable inner states. So the basis will now contain phenomenological facts about positive or negative feelings, just as Pautz recommends. These valence-facts will specify the particular sign and intensity of valence that is tied to a particular interpretable belief-typed state (e.g. per Lin, the affect is part of a wider state that includes the interpretable belief state in question). So the revised formulation is:

- I is the correct interpretation of a person *x* iff I is the interpretation that best rationalizes the way *x* is disposed to *act* given *x*’s *evidence,* and given the *valence* attached to the interpretable states that are typed as beliefs.

This extended basis for radical interpretation paves the way for the selection story: the correct interpretation is that one (or correct interpretations are those ones) which make the subject overall most rational. Credit is given for a belief-desire interpretation which makes structural sense of the agent’s choices. Credit is given for an interpretation which makes their beliefs responsive to the evidence; and (adds the anti-Humean) their desires reasonable. And credit is now also given for sustaining the structural rationality links between desire and positive and negative valence that I set out in the last section. In general, and in line with the model of interpretation we worked with earlier, structural rationality is of the first importance in determining which interpretation is most overall rational, but substantive rationality plays an important supplementary/tie-breaking role.

Return to Bob and Bob*. Let’s suppose, for the sake of argument, that we have an interpretation of Bob that makes him perfectly substantively and structurally reasonable. He believing as he ought (given his evidence), and his degrees of desire are as they ought to be—and these beliefs and desires rationalize his choice-dispositions. An interpretation that boosted the level of desire would still rationalize Bob’s choices, and requires no shift in his beliefs, but would make his desires unreasonable. Radical Interpretation in this way grounded a particular interpretation of Bob. Our trouble was, recall, that it would equally ground an interpretation of Bob* that attributes the same levels of desire (in line with the reasonable ones). But Bob* did not have those levels of desires, but systematically boosted ones. So the challenge was to explain this difference.

We now point to the following differentiating factor: Bob has affective valence attached to his beliefs which are *also* as they should be, matching the degrees of desire of the original interpretation. The original interpretation therefore still looks good, making Bob perfectly substantively and structurally rational. Bob*, by construction, has boosted affective valence, out of line with how it ought to be. And so there’s an inevitable mismatch. Attribute to Bob* desires that are as they ought to be, in his situation, and you interpret him as violating wide-scope norms of structural rationality linking affect and desire. Attribute to Bob* higher-level desires that satisfy those wide-scope norms, and you make his desires systematically less reasonable. But recall: the primary filter on interpretation are structural requirements. Giving priority to structural rationality, and only then looking to substantive rationality to shape our interpretation, tells us that the rationality-maximizing interpretation of Bob* is the second of the two mentioned.^20^

## Conclusion

By expanding the basis for radical interpretation to include valences, we’ve a solution to our puzzle. We have an inheritor of the toy theory of desire of Sect. 2—one that still assigns the structural rationalization of choice a central role. But structural rationality requires more, including the correct desire-valence links. Because valences are signed and come in interpersonally comparable intensities, we can vindicate that same sort of structure in intrinsic desire, and so in the non-intrinsic desires rationally connected, via belief, to intrinsic desire. Principles of substantive rationality—similarity to ideal desires, or values—can nuance these ascriptions, and will resolve potential indeterminacies in edge cases. But unlike the form of radical interpretation in Williams 2020, substantive rationality doesn’t play the central role here, and a good thing too since, as we saw, this would deliver incorrect results in our focal instances.

Our focal example of Bob/Bob* is, by design, an artificially simple case, where “all other things are equal”, and the signal from differences in affect comes through cleanly. In more ordinary cases—including the facts about comparative levels of desire in various propositions between you and me—there are many more factors in play. We’re yet to see whether the success can be sustained over *all* the cases, but I submit that the general sceptical *challenge* to comparability has gone, and gone in a way that coheres with the best clean data I know of.

## Footnotes

1 I see the argument for interpersonal incomparability as an indeterminacy challenge, and so see this as paralleling the debate over other content-focused indeterminacy problems for metasemantic theory, such as those of Putnam (1981) and Kripke (1982). Cf the reaction to Putnam’s “bomb” in Lewis (1984).

2 See, for example, List & Pettit (2011) and Bacharach (2006).

3 What follows is an informal gloss on some of the basic landscape in which social choice theory operates. See Gaertner (2009), passim.

4 See Gaertner, op cit,, for an accessible introduction to Arrow’s (1950) theorem, which shows any recipe for aggregating individual preferences must violate some intuitively compelling constraint. Arrow’s original “paradox” explored the prospects for aggregating *ordinal* preferences, and in order to engage with the possibility of cardinal preferences determining collective interest, one needs the more flexible framework developed in Sen (1970). The moral: we can escape from Arrow if and only if we have the right kind of interpersonal comparability.

5 Transformations of utilities that differ by (a) positive multiplication; and (b) addition of a constant are known as *positive affine transformations.* One question (pressed, for example, by Pareto 1906/1971) is whether we might not get the same result with any order-preserving transformation (e.g. squaring the utilities, taking logarithms). The answer, as Savage (1951) showed, is that if you transform utility in one of these broader ways, there will in general be no way to assign probabilities that make the probabilities and utilities/expected utilities fit together in structurally (“means-end”) rational ways (this is one consequence of Savage’s representation theorem). Affine transformations of utilities holding fixed probabilities will preserve means-end rational patterns, however. For an excellent account of these sort of results, see List, Christian (2003).

6 This case can be used to illustrate how a metaphysics of desire that allows for desire-comparisons may yet deliver the wrong comparisons. Suppose if Friday-Ann’s utilities are bounded, and so Ann*’s on Friday are too. One familiar way of introducing comparability is by identifying the levels of the respective topmost instantiated points, and respective bottommost instantiated points, of different people’s utility scales at every point in time. But this will end up identifying the strength of desire for running that Ann and Ann* have on Friday, contrary to the datum.

7 To state this more carefully: Bob and Bob* will choose in *correlated* ways in *correlated* decision situations. The caveats are needed if only because the outcomes to which feature in these decision situations will in one case involve Bob as agent, in another case, the distinct agent Bob*. Further, ex hypothesi the agents differ in their desire states, so there are qualitative differences between the outcomes in that respect. What’s really happening here is that there’s a bijection from a set of centred worlds involving Bob, to a set of centred worlds involving Bob* (and whatever qualitative differences that brings in train), such that where Bob has a certain proposition as evidence, and faces a set of options, Bob*’s evidence and options will be the image under the bijection of the evidence/options Bob has. The relevant notion of “having the same choice dispositions” is that when Bob is disposed to choose some option in one of his decision-situations, Bob* is disposed to choose the image of that option in the image under the bijection of that decision-situation. The observation is then that any probability-utility pair that rationalize Bob*’s choices will induce, via the bijection, a probability-utility pair that will rationalize Bob’s choices.

8 The two lines above speak of *the* correct interpretation and *the* interpretation which maximizes rationality. But what if two interpretations are tied at the top? We could revise the second line to speak of *a correct interpretation* tied to *interpretations which are at least tied for rationality-maximization.* The first line would then need revision in one of a number of ways—for example, it could be that a person desires that *p* to strength *d* only when all correct interpretations agree on this, fails to desire that *p* only when all correct interpretations agree on that, and that it is otherwise indeterminate whether the person has a desire for *p* of that strength. Alternatively, we might use a model of rationality which is *indeterminate* over total orders. “The most rational interpretation” would be indeterminate in reference, and such indeterminacy (on occasion) can filter up to indeterminacy in what a person believes or desires, with much the same effect. We don’t need to choose, for present purposes.

9 An indirect way is to appeal to (i) epistemic principles that fix the best-justified beliefs about what is good, given an agent’s evidence, and (ii) a desire-as-belief connection which tells us that rational agents align their degree of desire in *p* to their degree of belief that *p* is good. This raises many of the same issues as the direct route, and more besides.

10 There is an interesting connection here to explore between “most similar to the rational ideal” and “most rational”. See, for example, Julia Staffel’s (2019) exploration of the parallel connection in the case of belief. I’ve formulated the proposal here in a tie-breaking form, where coherence-like structural constraints are the lexically prior constraint on interpretation, and considerations of substantive rationality are lexically secondary. An alternative implementation allows some tradeoffs, so that a bit of structural rationality is worth giving up if it gains sufficiently more substantive rationality in interpretation.

11 Loophole: suppose the objective value facts in question were fundamentally *ordinal:* they tell you only that outcome A is better than outcome B, without in any way quantifying the degree of betterness. Accordingly, though the ideal agent has anti-Humean reason to prefer A to B, their desires being in accordance with these values gives us no grip on the kind of differences or levels of preference that the reason-responsive agent should exhibit. Only if the values themselves incorporate information about value-differences, levels, or signs, will these constrain desire-interpretation in a way relevant to the current dialectic.

12 Note that given unrestricted composition, there will be all sorts of objects that contain as proper parts phenomenal affect and a sensory state. But for the affect to be attached to the sensory state, the fusion of the two has to itself be a mental state, i.e., to have its own functional role in the subject’s psychology.

13 Compare Klein (2007) and Barlassina and Hayward (2019). These authors defend an *imperatival* account of the representational properties of affective states (aiming to give a representationalist account of the phenomenology thereby).

14 Compare Smithies & Weiss (2019), who argue for an interestingly related position: that a certain class of basic desires are simply dispositions to have affective experiences, and such desires give us reason for *action* only because this is so. The authors’ “affective experiences” are phenomenally conscious, intentional and valenced, and they include within this what we normally call “feelings of desire”: bodily appetites, pleasure and pain bound up with sensations, and emotions. These authors—and perhaps Pautz—may wish to resist the Feldman Barrett picture whereby affect is a fundamentally non-intentional phenomenal state that is generated by specific independently-characterisable intentional states. It's important, I think, to distinguish the narrow-scope principle that I’ve attributed to Pautz, and which would solve our puzzle, from the following narrow-scope principle: that there is reason to strongly desire that one undergoes *very positive affective experiences,* and reason to strongly desire that one doesn’t undergo very negative affective experiences. Anti-Humeans who are open to there being narrow-scope normative principles governing desire in the first place, i.e. there being (objective) reason to desire states of affairs characterized by justice, companionship, personal flourishing, can also consistently and plausibly add to this list *positively valenced affect.* But this does not establish a narrow-scope connection between positive affect attached to a thing, and intrinsic desire for that thing, as the Pautzian principles would. And unlike those principles, this variant would not, I think, help the interpretationist secure the right results in the Bob/Bob* case.

15 Though the ideology of affective states it uses is rather different from the two-dimensional circumplex model I’m presupposing, it’s striking that the computational model of affect developed by Reisenzein (2009) would be one which regulated affect, belief and desire in accordance with the above constraint.

16 I quite like this picture! But more would have to be said to justify the details of the thesis. For example, Kagan proposes a view on which you maximize well-being by enjoying the good to at least a certain level *k*, which tells us that from the point of view of maximizing well-being, we should take at least level *k* pleasure in the good. But Kagan does not think that there’s some kind of well-being *penalty* for taking *overmuch* pleasure in a good thing, and so on Kagan’s own view we can’t extract some exact level of appropriate pleasure to take in a given good thing. If, on the other hand, there is a principle that you’ve gone wrong if the strength of desire for a good thing is either too low or too high, relative to its value to you, then what we get out of the pair of principles is not the norm of matching laid out above, but a norm that says one ought to find *p* at least as desirable as the level of positive affect attached to the belief that *p*. As this variation makes clear, there is a lot to unpack and consider here, the details of which will inflect the overall story to be given. For now, I’ll stick with the version above as representative.

17 Note: this is perfectly consistent with thinking that one has narrow-scope reason to try to feel the strongly positive affect that was in the past associated with the suffering of others. And if you find that you could trigger this in a harmless way—say by playing violent video games—perhaps this gives you instrumental reason to indulge in that way. Thanks for a referee for pressing me to consider this case!

18 This sort of case introduces a place where individual differences can emerge. Some individuals would let the positive affect of the saving of the kingdom dominate their response, some would let the negative affect of the death of the general dominate their response, some would feel both simultaneously, and some would feel a kind of aggregated, muted positive response. Given what I have said so far, we can explain these by different patterns of attention: some would attend primarily to the killing of the general, triggering the negative affect. Some attend to the saving of the kingdom, and the positive affect. Some would hold both in mind, experiencing negative and positive affects simultaneously. The final obviously intelligible affective profile can also be explained, if we consider the intrinsic desirability for the monarch of the conjunction: general-being-killed-and-kingdom-saved. This will be an intrinsically desirable state of affairs for the monarch, but much *less* intrinsically desirable than the kingdom being saved, and so appropriately paired with a muted but positive affect. It may be that there’s an appropriate (or socially approved) way to distribute attention across the various states made salient by the news. Perhaps it reveals a particular kind of character to dwell on the good aspect, a different to dwell on the bad aspect, a third kind of character to hold both positive and negative news individually in focus, and a fourth to consider and respond to the whole information in aggregate. But all these are questions of how best to direct attention that I am silent on: they are all ways of conforming to the basic intrinsic-desire-to-affect connection.

19 I owe these cases to Matt Mandelkern.

20 We needn’t think that there’s complete lexical priority of perfect structural rationality. Perhaps *approximate* structural rationality across with respect to *most* base facts is what enjoys this status. More concessively, perhaps some structural irrationality may be admitted in the correct interpretation, if doing so buys a lot more substantive rationality, though I submit that the ‘rate of exchange’ here will be heavily weighted towards prioritizing structural rationality.
